# Angular and Rotational Lower Limb Alignment Variations in Children: Practical Guidance for Paediatricians

**DOI:** 10.3390/medicina62081499

**Published:** 2026-08-04

**Authors:** Josip Vlaic, Amelia Kruk, Marcin K. Wasko

**Affiliations:** 1 Division of Paediatric Orthopaedic Surgery, Children’s Hospital Zagreb, Ulica Vjekoslava Klaica 16, 10000 Zagreb, Croatia; jvlaic@yahoo.co.uk; 2 School of Medicine, University of Zagreb, 10000 Zagreb, Croatia; 3 Academy of Current Orthopaedics, 76-200 Slupsk, Poland; akruk@aorta.edu.pl; 4 Department of Radiology, The Centre of Postgraduate Medical Education, Marymoncka 99/103, 01-813 Warsaw, Poland; 5 Health Sciences Department, Ignacy Mościcki University of Applied Sciences in Ciechanów, Wojska Polskiego 51, 06-400 Ciechanow, Poland

**Keywords:** lower limb alignment, rotational deformity, angular deformity, in-toeing, genu varum, genu valgum, femoral anteversion, paediatric orthopaedics

## Abstract

*Background and Objectives:* Angular and rotational lower limb alignment concerns are a frequent reason for paediatric consultations. In most children, these findings reflect physiological developmental patterns that resolve spontaneously, but unilateral, progressive, or symptomatic cases may indicate pathology and require specialist assessment. *Materials and Methods:* This narrative review summarizes clinically relevant developmental norms, key elements of history and physical examination, common differential diagnoses, and practical indications for imaging and referral, with the objective of offering a structured clinical guidance framework for primary care and paediatric practitioners. *Results:* Physiological coronal plane lower limb alignment typically progresses from infant genu varum to neutral alignment by around 2 years, followed by transient genu valgum between 3 and 6 years, and stabilizes by approximately 7–8 years. Rotational alignment changes predictably with growth and most commonly presents as in-toeing due to femoral anteversion, internal tibial torsion, or metatarsus adductus. Out-toeing is less often physiological and should prompt careful evaluation for underlying disorders, including slipped capital femoral epiphysis. Clinical assessment should prioritize gait observation, foot progression angle, hip rotation, and thigh–foot angle, while imaging should be reserved for atypical presentations, asymmetry, progression, pain, or suspected systemic or neuromuscular disease. *Conclusions:* Most paediatric lower limb alignment “abnormalities” represent normal variants and require reassurance and observation. Recognizing age-appropriate patterns and red flags enables paediatricians to limit unnecessary interventions while ensuring timely referral and treatment for pathological conditions.

## 1. Introduction

Angular and rotational alignment abnormalities of the lower limbs are a frequent source of concern for parents, who often worry about their child’s function, appearance, and potential long-term consequences. In most cases, these findings represent physiological developmental patterns inherent to the natural maturation of the musculoskeletal system from birth through adolescence [1,2]. Children cannot be regarded as miniature adults, as lower limb alignment follows predictable, age-specific developmental trajectories that typically “normalize” spontaneously.

Epidemiological data confirm that lower limb variations are highly prevalent in early childhood. A study from one urban area reported knee deformities in over 50% of preschool-aged children and foot abnormalities in up to 85% [3]. However, another study found that more than two-thirds of children evaluated previously for lower limb alignment ultimately had physiological variants that improved with growth [4].

Despite the predominantly benign natural history, certain features warrant further evaluation. Unilateral presentation, asymmetry, progression over time, pain, limping, restricted range of motion, or associated neurological signs may indicate underlying pathology and require specialist referral [1,5]. The primary clinical challenge for paediatricians lies in distinguishing physiological variants from pathological deformities while avoiding unnecessary imaging or intervention.

Lower limb alignment differences may also influence psychosocial well-being. International studies have demonstrated that visible limb differences can restrict participation in recreational and social activities and negatively affect self-esteem [6,7]. Instruments such as the LIMB-Q Kids questionnaire provide structured tools for assessing these psychosocial domains and understanding the broader impact of alignment abnormalities [8].

The aim of this review is to provide practical, clinically oriented guidance for paediatricians in evaluating lower limb alignment variations, distinguishing physiological from pathological findings, and determining appropriate indications for observation, imaging, and referral. This narrative review summarizes current evidence based on a non-systematic literature search of PubMed, Scopus, and Web of Science databases, focusing on studies addressing physiological and pathological lower limb alignment in children, with particular emphasis on clinically relevant diagnostic and management strategies.

## 2. Materials and Methods

This narrative review was conducted to summarize current evidence regarding physiological and pathological angular and rotational lower limb alignment variations in children, with a focus on clinically relevant diagnostic and management strategies for paediatricians.

A non-systematic literature search was conducted between January and July 2026 using PubMed, Scopus, and Web of Science to identify publications relevant to lower limb alignment, rotational deformities, gait assessment, and common paediatric orthopaedic conditions. The same combinations of search terms were applied across all three databases, with minor modifications to accommodate database-specific search syntax where necessary. Additional landmark publications, clinical practice guidelines, and reference lists of key articles were reviewed to ensure comprehensive coverage of clinically important topics.

Titles and abstracts identified through the database search were screened for relevance. Full-text articles considered relevant to the objectives of this review were subsequently assessed. Studies were selected based on their clinical relevance, with particular emphasis on publications describing physiological development, clinical examination, differential diagnosis, imaging, and management of lower limb alignment variations in children.

The search primarily included articles published in English from January 2000 to March 2026. Earlier landmark studies were included when considered fundamental for understanding the physiological development of lower limb alignment or widely accepted clinical examination methods. Search terms included combinations of: “lower limb alignment”, “angular deformity”, “rotational deformity”, “genu varum”, “genu valgum”, “femoral anteversion”, “tibial torsion”, “metatarsus adductus”, “in-toeing”, “out-toeing”, “paediatric orthopaedics”, “child”, and “gait”.

Priority was given to clinical practice guidelines, systematic reviews, narrative reviews, observational studies, and seminal publications describing normal developmental alignment, clinical examination techniques, differential diagnosis, and treatment recommendations. Case reports, studies involving adult populations only, non-English publications, and studies not directly relevant to the objectives of this review were generally not included unless they represented landmark descriptions of specific conditions.

The evidence was synthesized narratively, with particular emphasis on practical clinical application in primary care and paediatric practice rather than quantitative comparison of study outcomes.

## 3. Diagnosis and Management of Lower Limb Deformities

Evaluation of a child with a lower limb alignment abnormality should begin with a detailed history, including birth history, neonatal hip ultrasound report, motor development, age of onset, and family history of similar conditions. It is essential to establish whether the abnormality is progressive or symmetrical, and whether additional symptoms such as pain, restricted range of motion, or neurological signs are present. The key elements of the medical history that should be assessed during the initial evaluation are summarized in Table 1.

**Table 1:** Pertinent history for children presenting with lower extremity alignment or rotational abnormalities.

History Element	Key Questions	Age-Based Diagnostic Clues	Red Flags Requiring Referral	Possible Associated Conditions
Age at onset	When did the parent first notice the abnormal gait or limb alignment?	Infancy: metatarsus adductus; Toddler: internal tibial torsion; Age 3–6: femoral anteversion; Adolescence: consider slipped capital femoral epiphysis (SCFE)	Onset after 8 years or sudden onset in adolescence	Metatarsus adductus, tibial torsion, femoral anteversion, SCFE
Progression over time	Is the deformity improving, stable, or worsening?	Physiological deformities typically improve with growth	Progressive deformity or worsening alignment	Blount disease, rickets, physeal arrest
Laterality	Is the deformity unilateral or bilateral?	Physiological variants are usually symmetrical	Persistent unilateral deformity	Blount disease, growth plate injury, neuromuscular disorder
Pain	Does the child report pain in the hip, thigh, knee, or foot?	Physiological torsional variants are usually painless	Any persistent pain, especially hip or knee pain	SCFE, infection, inflammatory disease
Limping or gait disturbance	Does the child limp, trip frequently, or have difficulty running?	Mild clumsiness common with femoral anteversion	Limp or sudden gait change	SCFE, neuromuscular disorders, structural deformity
Functional limitations	Does the condition limit sports participation or daily activities?	Most physiologic variants do not limit function	Functional impairment affecting activity	Severe torsional deformities, neuromuscular disease
Birth history	Were there complications during pregnancy or delivery? Breech presentation? Prematurity?	Intrauterine positioning may cause early foot deformities	Associated congenital abnormalities	Metatarsus adductus, developmental dysplasia of the hip
Developmental milestones	Did the child achieve sitting, crawling, and walking milestones on time?	Normal milestones suggest benign torsional variation	Developmental delay or regression	Cerebral palsy, neuromuscular disorders
Family history	Do family members have similar limb alignment patterns?	Familial patterns common in femoral anteversion	Family history of skeletal dysplasia	Familial torsional variants, skeletal dysplasias
Sitting habits	Does the child frequently sit in a “W” position?	Common in children with femoral anteversion	Associated hip stiffness or pain	Femoral anteversion
Previous trauma or infection	Has the child had fractures, infections, or surgery involving the lower limbs?	May explain asymmetrical deformity	History of physeal injury	Post-traumatic deformity, growth arrest
Growth pattern	Has the child shown normal growth and height progression?	Normal growth typical for physiologic variants	Short stature or abnormal growth pattern	Rickets, skeletal dysplasia
Systemic symptoms	Are there symptoms such as fatigue, weakness, or metabolic abnormalities?	Usually absent in benign alignment variants	Systemic illness or metabolic abnormalities	Rickets, endocrine disorders
Parental concern	What specifically concerns the parents—appearance, gait, or function?	Cosmetic concerns common in physiological variants	Rapid change in appearance or function	Pathological deformity requiring evaluation

### 3.1. Age-Specific Differential Diagnosis of Hip Disorders

Hip pathology should always be considered during the evaluation of children presenting with gait abnormalities, rotational deformities, or apparent lower limb malalignment. Although many alignment variations are physiological and resolve spontaneously with growth, disorders affecting the hip may alter gait patterns, limb positioning, and lower extremity alignment, thereby mimicking primary deformities of the lower limbs. Careful assessment of hip range of motion, limb length, gait characteristics, and the presence of pain is therefore an essential component of the clinical examination.

The differential diagnosis of hip disorders varies according to the child’s age. In infants, developmental dysplasia of the hip (DDH) should be considered, particularly in the presence of limited hip abduction, apparent leg-length discrepancy, pelvic asymmetry, or delayed motor development. In school-aged children, Legg–Calvé–Perthes disease typically presents with a painless limp or intermittent hip, groin, or referred knee pain accompanied by restricted hip motion, especially abduction and internal rotation. In adolescents, SCFE is an important cause of new-onset out-toeing gait, hip or knee pain, and decreased internal rotation, requiring urgent orthopaedic referral. Recognition of these age-dependent conditions facilitates timely diagnosis, appropriate imaging, and early specialist management [9]. The age-specific differential diagnoses are summarized in Table 2.

**Table 2:** Age-specific differential diagnoses in children presenting with gait abnormalities or apparent lower limb malalignment.

Age	Common Conditions Presenting with Lower Limb Malalignment
Infant (0–12 months)	DDH, metatarsus adductus
Toddler (1–3 years)	internal tibial torsion, residual DDH
Preschool child (3–5 years)	Increased femoral anteversion
School-aged child (5–10 years)	Legg–Calvé–Perthes disease
Adolescent (10–16 years)	SCFE

Legg–Calvé–Perthes disease should be considered in school-aged children, typically between 4 and 8 years of age, presenting with limping, hip or referred knee pain, or progressive gait abnormalities. Clinical examination often reveals restricted hip abduction and internal rotation, while pain may be absent during the early stages of the disease. As delayed diagnosis may lead to femoral head deformity and poorer long-term outcomes, children with suspected Legg–Calvé–Perthes disease should be referred promptly for orthopaedic assessment and appropriate imaging [10,11].

### 3.2. Physical Examination

Physical examination should be performed in both standing and supine positions. Key components include:

• Observation of gait and foot positioning,
• Foot progression angle (FPA),
• Hip rotation (assessed prone with knees flexed),
• Thigh–foot angle (TFA),
• Frontal plane alignment (genu varum/valgum),
• Symmetry and leg length,
• Presence of foot abnormalities (e.g., metatarsus adductus).

### 3.3. Measurement Techniques

The physical examination of a child presenting with a lower limb alignment abnormality should be comprehensive and performed under both dynamic and static conditions. Assessment in standing, supine, and prone positions allows for differentiation between deformities originating from the femur, tibia, or foot. A systematic evaluation of rotational and angular parameters is essential to accurately identify the anatomical level of the deformity and to distinguish physiological variations from pathological conditions [11,12,13,14].

Gait observation constitutes the initial and integral component of the examination. The child should be assessed while walking barefoot along a straight path, with careful observation from anterior, posterior, and lateral perspectives. Particular attention should be paid to the direction of foot progression, symmetry of step length, and the presence of compensatory movements. This dynamic assessment provides an overall functional impression of lower limb alignment and reflects the combined rotational contributions of the femur, tibia, and foot [12,13,14,15].

The FPA is subsequently evaluated during gait and represents the angular relationship between the longitudinal axis of the foot and the line of progression. This parameter may be estimated clinically or measured more precisely using video-based gait analysis. A positive FPA indicates external rotation, whereas a negative value reflects internal rotation. Importantly, FPA serves as a composite measure and does not allow for differentiation between femoral, tibial, or foot-related causes of rotational deviation [11,12,13,14,15].

To further delineate the origin of rotational abnormalities, hip rotation should be assessed with the patient in the prone position and the knees flexed to 90 degrees. The examiner passively rotates the lower leg, which translates into internal and external rotation at the hip joint. These movements are quantified using a goniometer. Increased internal rotation combined with reduced external rotation is suggestive of increased femoral anteversion, whereas the opposite pattern may indicate femoral retroversion. This assessment is critical for distinguishing proximal rotational deformities [11,12,13,14,15].

Evaluation of tibial torsion is performed by measuring the TFA also with the patient in the prone position and the knee flexed to 90 degrees [16]. The angle between the longitudinal axis of the thigh and the axis of the foot is determined. A positive TFA indicates external tibial torsion, while a negative value reflects internal tibial torsion [15]. Unlike FPA, TFA provides a more isolated assessment of tibial rotational alignment and is therefore considered one of the most reliable clinical parameters for evaluating tibial torsion [12].

Assessment of frontal plane alignment is conducted with the patient in a standing position. In cases of suspected genu varum, the ankles are positioned together, and the distance between the medial femoral condyles is measured [12,17]. Conversely, in genu valgum, the knees are approximated, and the distance between the medial malleoli is recorded. These measurements allow for quantification of coronal plane deformities and facilitate the identification of physiological versus pathological angular deviations [18].

Symmetry and limb length should also be evaluated to exclude discrepancies that may contribute to abnormal alignment or gait patterns. True limb length is measured from the anterior superior iliac spine to the medial malleolus, while apparent limb length is assessed from the umbilicus to the medial malleolus [11,19]. Additional clinical tests, such as the Galeazzi test, may be employed to detect femoral shortening or asymmetry. Distinguishing between true and apparent discrepancies is essential for accurate diagnosis and management [11,20].

**Figure 1:**
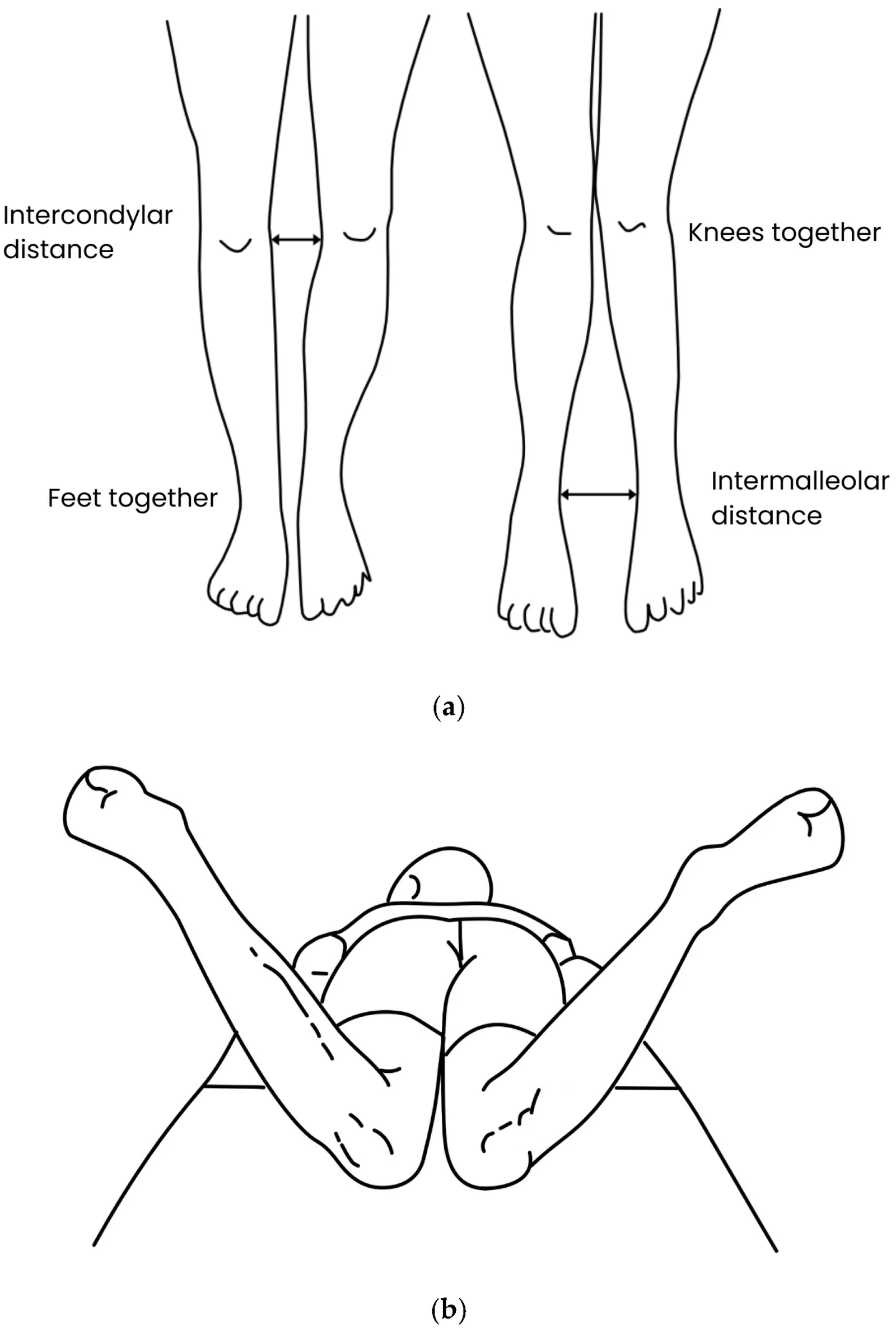

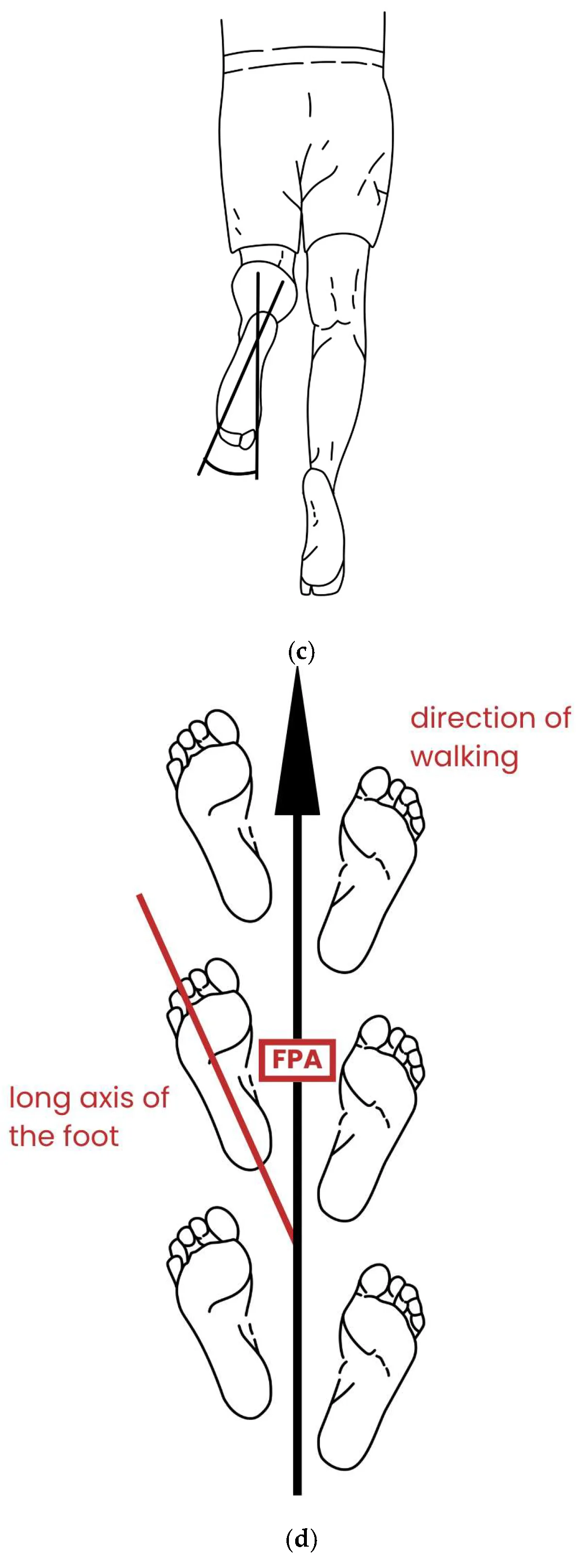
Clinical examination techniques for assessment of lower limb alignment in children: (**a**) Intercondylar and intermalleolar distance; (**b**) thigh-foot angle; (**c**) hip rotation—internal rotation; (**d**) foot progression angle.

Finally, the examination should include a detailed assessment of foot morphology to identify distal contributors to rotational abnormalities. The heel bisector method is commonly used, in which a line drawn through the longitudinal axis of the heel is extended distally toward the forefoot. In a normally aligned foot, this line passes between the second and third toes. Deviation of this line laterally suggests metatarsus adductus. Assessment of flexibility is also important to differentiate between flexible and rigid deformities [11,21]. The key components of the physical examination in children with lower extremity alignment or rotational abnormalities are summarized in Table 3. The principal clinical examination techniques used to assess lower limb alignment and rotational abnormalities are illustrated in Figure 1.

Although clinical examination remains the cornerstone of evaluating lower limb alignment in children, all measurements are inherently examiner-dependent and should be interpreted in the context of the child’s age, overall development, and clinical presentation [4,11]. Interobserver variability has been reported for several rotational measurements, particularly hip rotation and thigh–foot angle, emphasizing the importance of standardized examination techniques and repeated assessments during follow-up [12,15,22]. Furthermore, the foot progression angle represents a composite measure reflecting the combined rotational contribution of the femur, tibia, and foot rather than an isolated anatomical parameter [13,14,15]. Consequently, no single clinical measurement should be interpreted in isolation [4]. When findings are inconsistent, asymmetrical, progressive, associated with pain or functional limitation, or when the anatomical origin of the deformity remains uncertain, appropriate imaging studies should be considered to confirm the diagnosis and guide further management [5,9,11]. Integrating clinical examination with age-specific developmental norms remains the most reliable approach for distinguishing physiological variants from pathological conditions. Serial clinical assessment is often more informative than a single isolated examination, particularly in young children with physiological developmental variations [13,14].

#### Imaging

Imaging is indicated only in cases of suspected pathology (e.g., Blount’s disease, DDH, SCFE, rickets), lack of improvement, or unilateral deformities. Imaging modalities should be selected according to the patient’s age and the suspected diagnosis. Hip ultrasonography is the preferred imaging modality for infants with suspected DDH, whereas anteroposterior (AP) pelvic radiographs are recommended in older children after ossification of the femoral head. Standard radiographs in two planes or long-leg standing radiographs are usually the first-line studies for evaluating lower limb deformities. Advanced imaging (MRI or CT) should be reserved for well-defined indications, including suspected physeal arrest, SCFE, or complex skeletal dysplasia [1,5,23].

For paediatricians, the primary role is to determine whether the clinical course follows the expected physiological pattern and to reassure parents when findings are within normal limits. When pathology is suspected, referral to a paediatric orthopaedist is required. The orthopaedic surgeon is responsible for further imaging, diagnosis, and treatment planning.

## 4. Angular Deformities

Angular alignment variations refer to deviations in the coronal plane axis of the lower limb, resulting in knees positioned closer together (genu valgum—knock knees) or farther apart (genu varum—bowlegs). A characteristic developmental sequence is observed: physiological bowlegs in newborns and infants, progressing to neutral alignment by age 2, followed by transient genu valgum between ages 3 and 6. By the ages of 7-8 years, most children attain a physiological alignment with mild valgus of approximately 5–7 degrees [24,25,26].

In clinical practice, angular alignment can be assessed using simple bedside measurements. Intercondylar distance, measured with the medial malleoli in contact (or more formally with the ankles together), is used to estimate genu varum, whereas intermalleolar distance, measured with the knees touching, is used to assess genu valgum. In physiologically developing children, these distances remain small and decrease with age; values exceeding age-appropriate ranges or the presence of asymmetry should prompt further evaluation [5]. From a biomechanical perspective, lower limb alignment is defined in relation to the mechanical axis, which extends from the center of the hip joint to the center of the ankle joint. A knee positioned along this load-bearing axis is considered neutrally aligned [27].

Most children remain within normal developmental limits and do not require intervention [28]. However, the presence of asymmetry, lack of improvement, unilateral alignment abnormalities, or suspicion of metabolic or developmental disorders (e.g., Blount’s disease, rickets, bone dysplasias) warrants further diagnostic evaluation and referral to a specialist. In most cases, careful clinical evaluation is sufficient, while imaging should be reserved for atypical or pathological presentations [1,5,7].

### 4.1. Genu Varum (Bowlegs)

Physiological bowlegs are considered normal until age 2 [11,14]. Infants may demonstrate up to 15 degrees of varus, which typically resolves by the second year of life. Newborns usually present with a pronounced physiological varus; however, if disproportionately short limb length is observed, achondroplasia or hypochondroplasia should be suspected [29]. In these patients, birth length may be normal, and the first clinical clue may be persistent varus in a 2.5-year-old child with short stature [30]. Children with persistent deformity after this age should also be carefully monitored for rickets as a potential underlying pathology [31,32].

RED FLAG

Blount disease (tibia vara) should be suspected in children presenting with persistent or progressive varus beyond the age of 2–3 years, particularly when asymmetrical. Early-onset (infantile) and adolescent forms differ in presentation and progression. Clinical examination frequently demonstrates marked internal tibial rotation and a varus thrust during gait, reflecting dynamic instability of the knee during the stance phase. Radiographic findings typically demonstrate medial proximal tibial physeal disturbance with metaphyseal beaking. Delayed diagnosis may result in progressive deformity requiring complex surgical correction.

**Figure 2:**
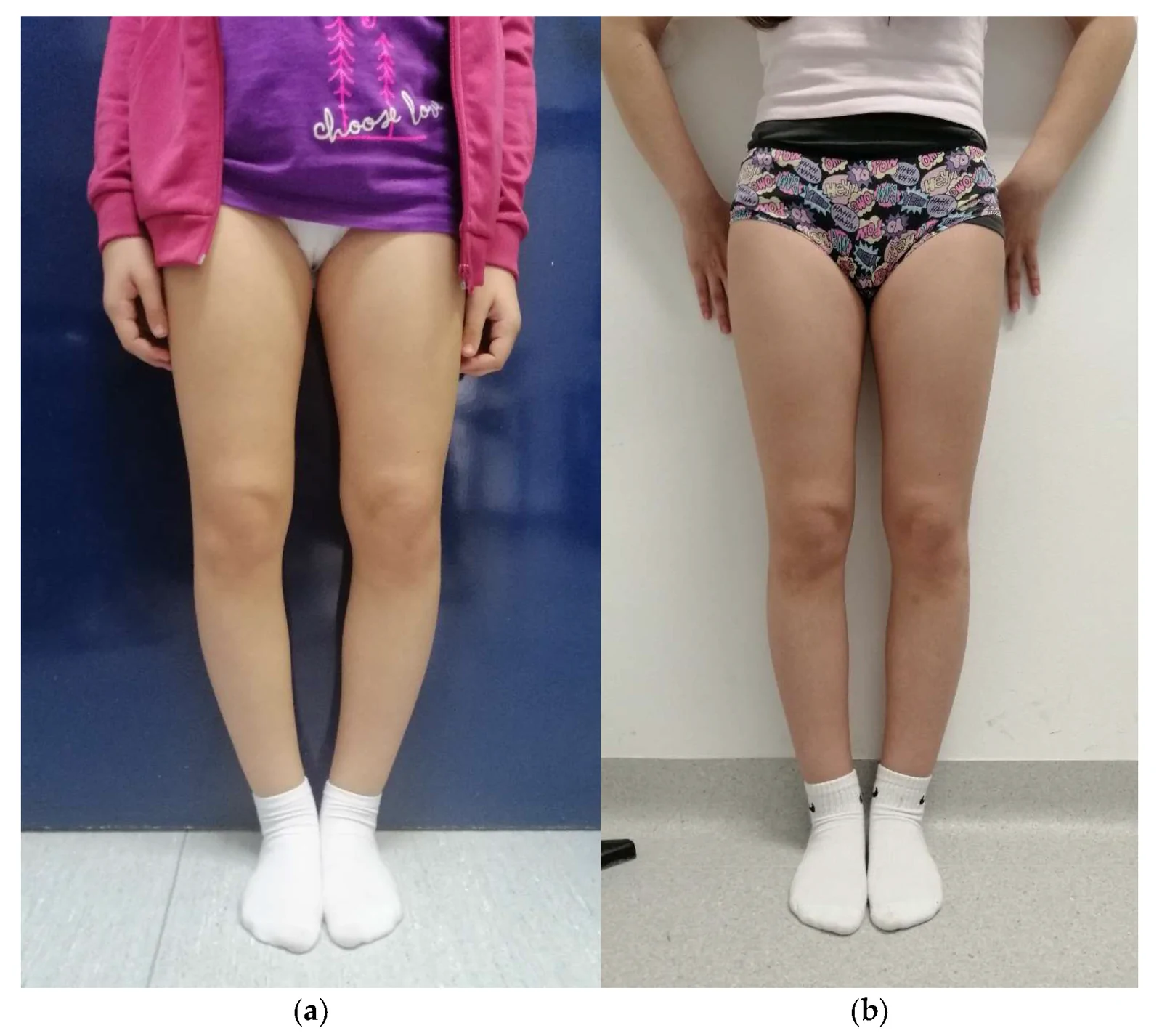
(**a**) Idiopathic varus knees in a 10-year-old girl. Due to open physes and remaining growth potential, a surgical procedure that involved growth modulation (hemiepiphysiodesis) was performed. (**b**) One year after surgery, knee alignment is correcting.

Some children develop persistent varus deformity despite a normal family history, open growth plates, and normal metabolic profiles. These cases are classified as idiopathic bowlegs and are often referred to an orthopaedic surgeon due to cosmetic concerns (Figure 2).

Depending on the patient’s age, remaining growth potential, and the severity of the deformity, growth modulation using hemiepiphysiodesis may be considered [1,5,33]. In older adolescents with inactive growth plates, correction of the mechanical axis of the lower limb requires osteotomy [1,5]. Although knee osteotomy for correction of excessive varus deformity in children has been used in cases of rickets, Blount’s disease, epiphyseal and metaphyseal injuries, in healthy adolescents with excessive varus deformity, the main indication for osteotomy is more than two standard deviations (2SD) of varus malalignment [34].

RED FLAG

In cases of suspected rickets, clinical features such as widened wrists, delayed growth, hypotonia, or biochemical abnormalities (vitamin D deficiency, hypocalcaemia, elevated alkaline phosphatase) should prompt laboratory investigation.

**Figure 3:**
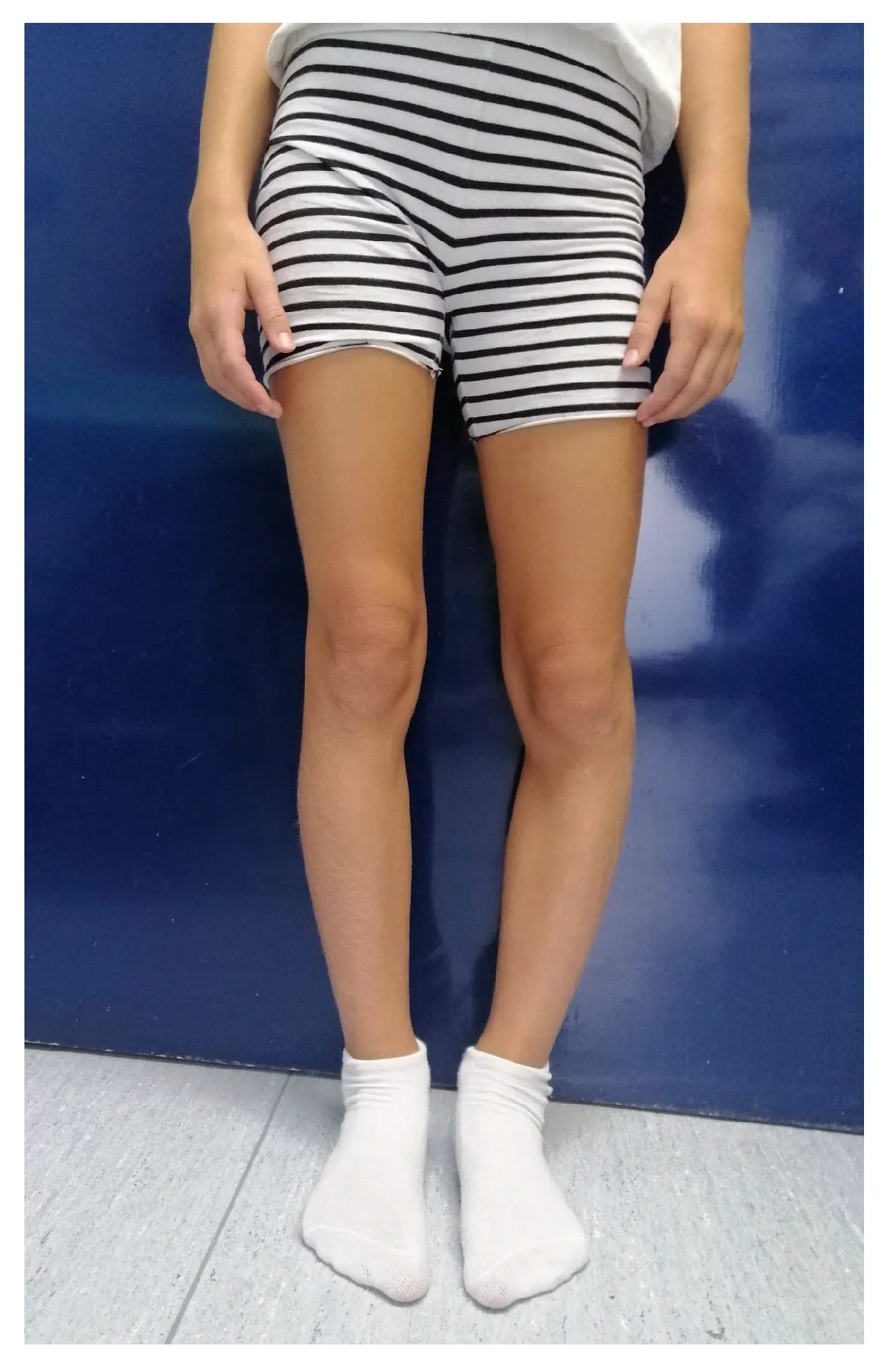
Eleven-year-old girl with left-sided adolescent Blount disease. A significant varus deformity is present.

Unilateral, progressive, or asymmetrical varus deformities may suggest Blount’s disease or other conditions such as partial growth arrest secondary to fracture, infection, or physeal bar (Figure 3) [28,30].

In such cases, surgical treatment may be the only option. In practice, clinical evaluation and observation are usually sufficient in physiological cases, whereas radiographs are indicated for atypical, unilateral, or progressive deformities.

### 4.2. Genu Valgum (Knock Knees)

**Figure 4:**
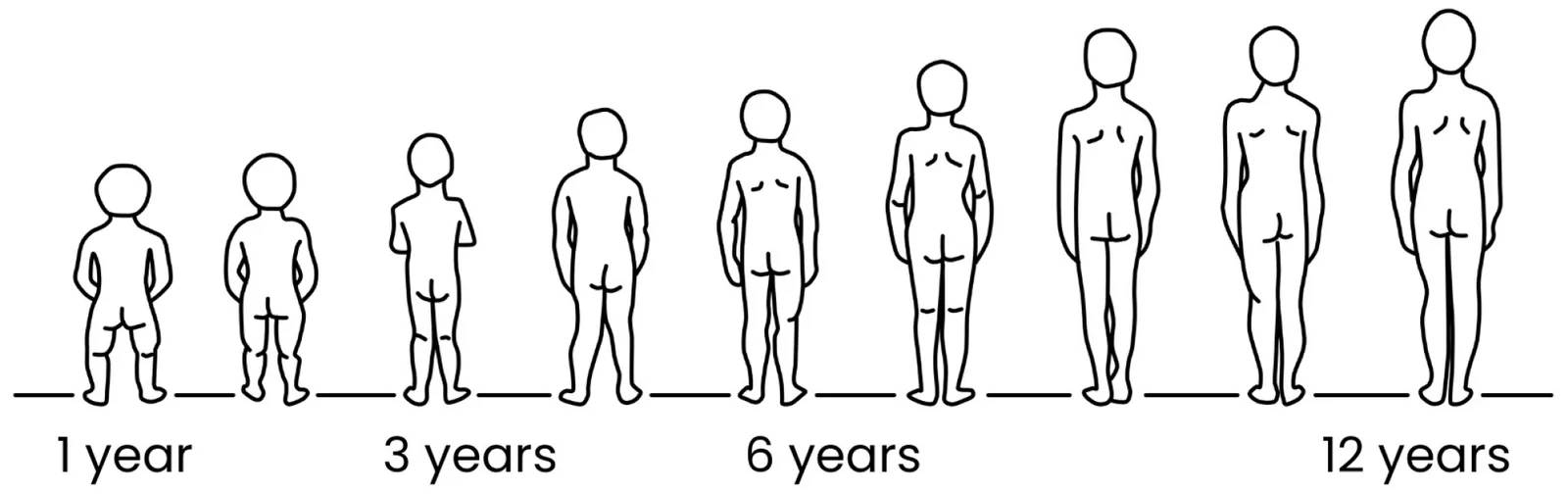
Physiological development of lower limb alignment in the coronal plane from age 1 to 12 years.

Physiological genu valgum is typically observed between the ages of 3 and 6 years, sometimes reaching up to 12–13 degrees, and usually resolves by the age of 7 to 8 years (Figure 4) [4,5,34].

Marked deviations from this pattern, particularly unilateral cases, warrant further diagnostic evaluation (e.g., radiographs, hormonal testing). Newborns with bone dysplasias often have a characteristic appearance; therefore, suspected cases should be investigated.

Children with skeletal dysplasias often present with characteristic phenotypic features, including disproportionate short stature, rhizomelic or mesomelic limb shortening, and associated skeletal abnormalities such as joint laxity or deformities of the spine and chest [11,35]. In conditions such as Morquio disease, valgus deformity is frequently accompanied by ligamentous laxity and hip dysplasia, whereas Ellis–van Creveld syndrome is associated with short limbs, polydactyly, and thoracic abnormalities. Recognition of these characteristic features during clinical examination facilitates early diagnosis and appropriate referral [16].

Recent studies also suggest that increased body mass index may be associated with a higher prevalence of genu valgum in children, possibly due to altered mechanical loading across the knee joint [36,37,38].

Similar to varus knees, genu valgum can also be idiopathic, meaning there is no underlying disease causing the deformity. In these cases, if growth potential remains, growth plate modulation with hemiepiphysiodesis may be performed, correcting the deformity with a very low-risk surgical procedure. For adolescent patients with persistent genu valgum, an osteotomy is indicated. Main knee problems in these cases that need to be corrected are abnormal gait and functional disturbances such as difficulty in running, knee discomfort, patellar malalignment, lateral patellar instability, and ligamentous instability [39].

**Figure 5:**
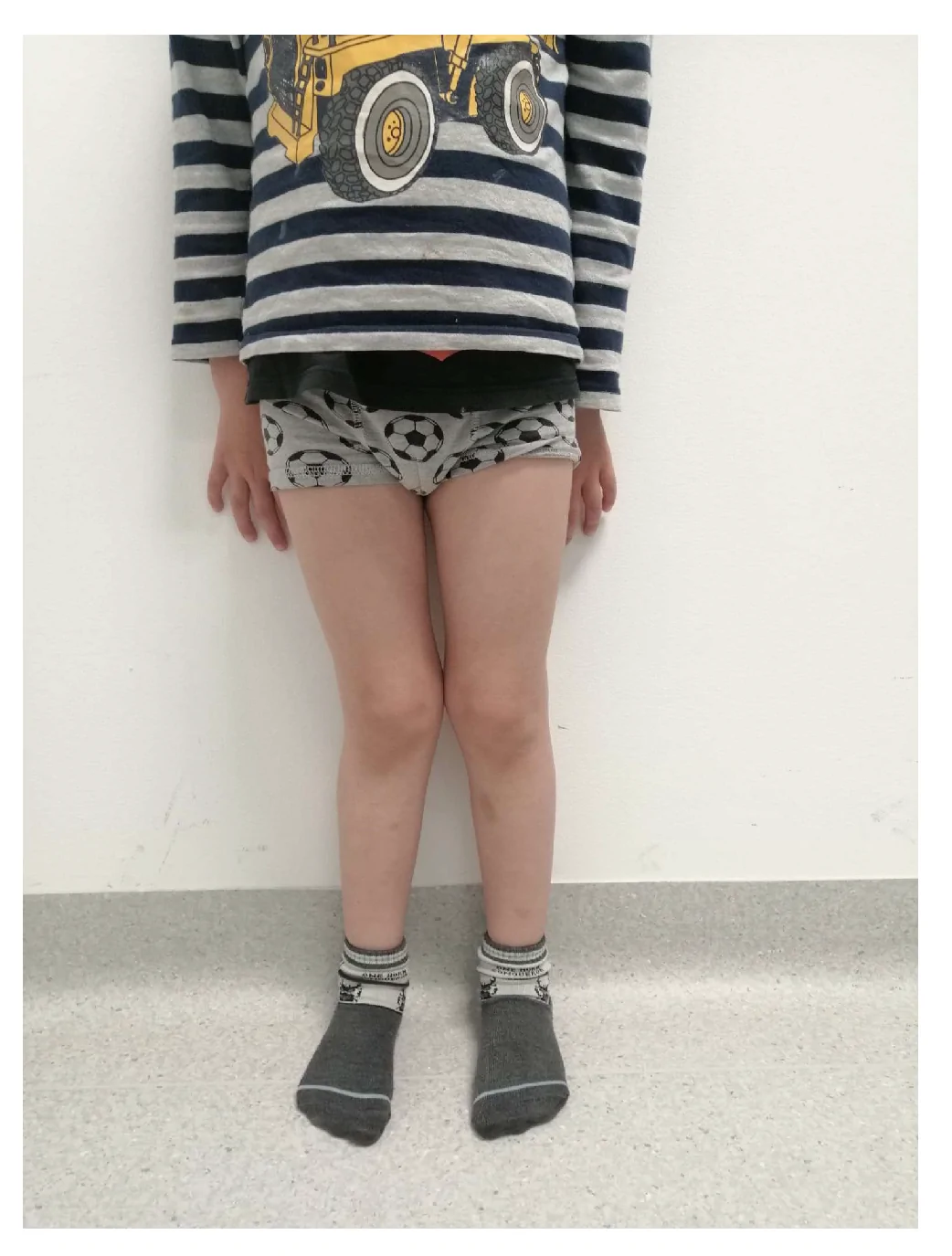
Cozen phenomenon in a four-year-old child. One year after sustaining a proximal tibial metaphyseal fracture on the right side, moderate valgus deformity is observed.

In a specific situation, following a proximal tibial metaphyseal fracture, a Cozen phenomenon may occur, presenting as unilateral genu valgum (Figure 5).

Therefore, in children aged 2 to 8 years with such fractures, parents should be informed of this potential outcome. The exact mechanism of tibial valgus development after this injury remains unclear; however, factors such as improper reduction, periosteal entrapment at the fracture site, or asymmetrical medial tibial overgrowth are suspected. In most cases, the deformity is mild and resolves spontaneously over time [39]. Nevertheless, all children presenting with asymmetric lower limb deformity following a proximal tibial fracture should be evaluated by an orthopaedic surgeon to exclude progressive or pathological valgus [40].

### 4.3. Summary of Angular Deformities

Angular deformities encompass both genu varum and genu valgum and represent a normal part of musculoskeletal development. Most children require only observation and reassurance. However, unilateral, persistent, or progressive deformities should prompt further evaluation and possible referral to a paediatric orthopaedic surgeon.

## 5. Rotational Deformities

Rotational deformities refer to deviations in the horizontal—transverse plane, i.e., the rotational axis of the lower limbs, involving femoral (thigh bone) torsion, tibial (shin bone) torsion, and forefoot alignment. In childhood, these variations most commonly manifest as in-toeing or out-toeing gait. In the majority of cases, they are physiological and tend to resolve spontaneously with skeletal maturation [1,2,41].

### 5.1. Physiological Development

Rotational alignment changes in a predictable manner throughout growth [26]. Newborns typically demonstrate increased femoral anteversion of up to 40 degrees, which gradually decreases to approximately 15 degrees in young adulthood [11]. Tibial torsion shifts from negative values (internal rotation) in infancy to positive values (external rotation) by the age of 5 years [1,2].

### 5.2. Clinical Features and Diagnosis

In most cases, rotational variations are symmetrical and do not impair mobility. Clinical assessment should include evaluation of hip rotation, thigh–foot angle (TFA), foot progression angle (FPA), and gait observation. In clinical practice, these measurements are typically obtained during the torsional profile examination [12,13]. The foot progression angle is assessed during gait by observing the angle formed between the long axis of the foot and the direction of walking. Internal rotation of the hip is evaluated with the child in the prone position and knees flexed to 90°, allowing assessment of femoral torsion. The thigh–foot angle is measured with the child prone and the knee flexed, comparing the thigh and foot axes to estimate tibial torsion. Measurements that fall substantially outside expected age-related ranges may indicate pathological rotational deformity and should prompt further evaluation. Age-specific reference values for rotational parameters are summarized in Table 4.

**Figure 6:**
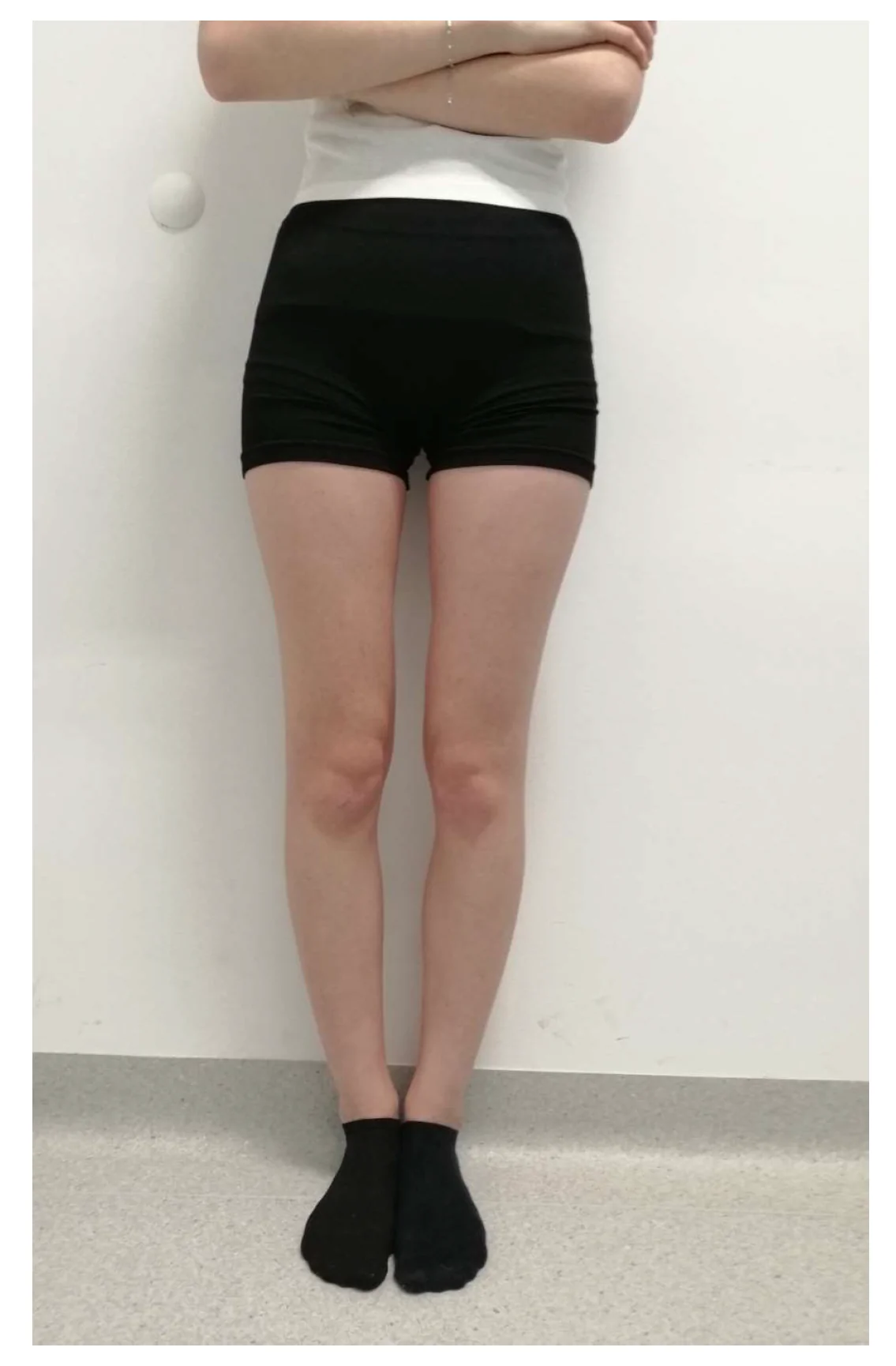
“Squinting patellae” appearance: both patellae point inward when the patient is standing with the feet facing forward.

Excessive femoral anteversion and/or external tibial torsion may also present as the characteristic “squinting patellae” appearance (Figure 6).

### 5.3. In-Toeing

The likely cause of in-toeing often correlates with the child’s age at presentation [11]. Metatarsus adductus is typically identified in infancy, internal tibial torsion commonly becomes evident in toddlers, and increased femoral anteversion is most frequently observed in children between 3 and 6 years of age [12].

The three most common causes of in-toeing are femoral anteversion, metatarsus adductus (forefoot adduction), and internal tibial torsion [1,2,23].

Femoral anteversion is the most frequent cause [13]. It may reach up to 40 degrees in newborns and decreases to about 15 degrees in young adulthood. Clinical features include a negative foot progression angle, excessive internal hip rotation (>70 degrees), and frequent association with W-sitting. Most cases resolve spontaneously by age 8. Conservative measures such as orthotics, wedges, or special shoes do not alter the natural history and may have negative psychological effects [1,2,42].

**Figure 7:**
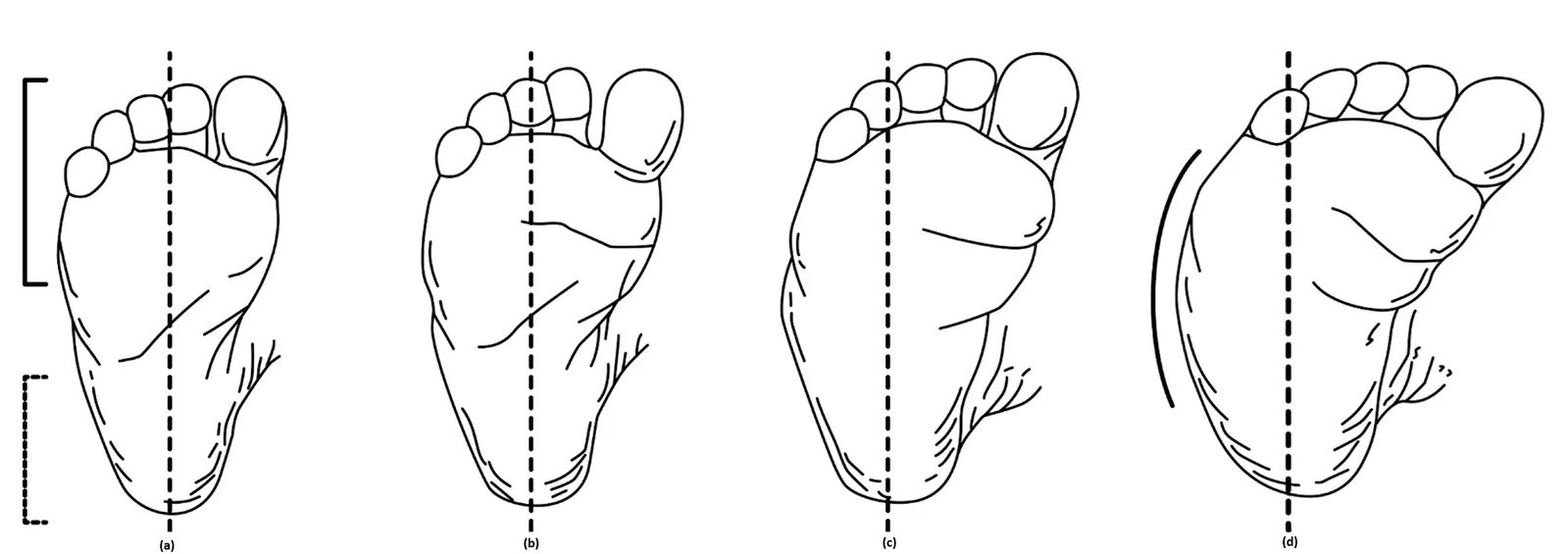
Classification of metatarsus adductus severity based on the heel bisector line method. From left to right: (**a**) normal foot—heel bisector line passes through the second toe; (**b**) flexible deformity—line passes through the third toe, correctable beyond the midline with gentle pressure; (**c**) semi-flexible deformity—line passes through the fourth toe, correctable to the midline only; (**d**) rigid deformity—line passes through the fourth–fifth toe, not passively correctable, may require orthopaedic management.

Metatarsus adductus is characterized by adduction of the forefoot relative to the rearfoot, producing a curved lateral border of the foot, often described as “banana-shaped” [23]. In most cases, the deformity is flexible and correctable with gentle pressure; in rigid cases, refer to a paediatric orthopaedist. Clinical assessment often includes evaluation of the heel bisector line. In this method, an imaginary line is drawn through the midpoint of the heel and extended distally across the forefoot. In normally aligned feet, this line passes through the second toe. Deviation toward the lateral toes indicates increasing severity of metatarsus adductus: flexible, semi-flexible, and rigid. Flexible deformities can be corrected beyond the midline and usually resolve spontaneously without intervention. Semi-flexible deformities can be corrected to the midline but not beyond it, while rigid deformities cannot be passively corrected and may require orthopaedic management, including casting or surgical consultation in selected cases [21]. The classification of deformity flexibility and corresponding clinical findings are illustrated in Figure 7.

Internal tibial torsion is a physiological finding in newborns, with an average thigh–foot angle (TFA) of −5 degrees. With growth, this progresses to approximately +10 degrees by age 10 and +25 degrees in adolescence [10]. Failure of this transition may result in persistent in-toeing, although this is rare [13].

### 5.4. Out-Toeing

The main causes of out-toeing include:

• Physiological external rotation contracture of the hip (infancy),
• External tibial torsion,
• Femoral retroversion (less common),
• Iliotibial band contractures,
• Acquired disorders (e.g., SCFE) [1,2,20].

External rotation contracture of the hip is a common physiological finding during infancy, resulting from intrauterine positioning. Infants typically demonstrate increased external rotation and limited internal rotation of the hip, which may contribute to an out-toeing gait when they begin standing and walking. This condition usually improves spontaneously during the first years of life as the hip capsule gradually stretches and normal hip rotation develops [11,13,28]. Persistence beyond early childhood, asymmetry, or association with pain or restricted hip motion should prompt further evaluation for underlying pathology [13,28].

Out-toeing is less commonly physiological than in-toeing and should raise greater suspicion of pathology [13]. Tibial torsion has been shown to evolve with age, with evidence of continued rotational remodeling into adulthood, and excessive external tibial torsion has been implicated as a contributing factor to anterior knee and patellofemoral pain [43,44]. Femoral retroversion is rare but can cause hip and knee pain when pronounced [13,28].

The most serious cause of out-toeing gait is SCFE, which represents displacement through the proximal femoral physis [45,46]. It most commonly occurs in early adolescence and may initially present with subtle symptoms. Clinical features include limp, hip or referred knee pain, reduced internal rotation of the hip, and obligate external rotation during passive flexion. Progressive external foot progression may be the first visible sign. Any adolescent presenting with new-onset out-toeing accompanied by pain or restricted hip motion should be considered to have SCFE until proven otherwise [45,47]. Prompt radiographic evaluation and urgent orthopaedic referral are mandatory to prevent complications such as avascular necrosis or early degenerative changes.

### 5.5. Associated Foot Conditions

In addition to rotational variations, paediatricians frequently encounter physiologic flexible flatfoot (pes planus) during evaluation of gait abnormalities. This condition is common in early childhood due to ligamentous laxity and incomplete development of the medial longitudinal arch [48]. Most cases are asymptomatic and resolve spontaneously with growth [48,49]. In the absence of pain, stiffness, or functional limitation, investigation or treatment is not required. Rigid flatfoot or persistent painful deformities should prompt evaluation for underlying conditions such as tarsal coalition [10,48].

Flexible flatfoot is a common finding in infants and young children and is considered a physiological developmental variant. Flexible flatfoot is considered physiological until approximately 6–8 years of age, as the medial longitudinal arch gradually develops during normal growth. Therefore, most asymptomatic children do not require treatment, and observation with parental reassurance is generally sufficient.

Symptomatic flexible flatfoot, characterized by activity-related pain, fatigue, or functional limitations, may warrant conservative management, including activity modification, stretching exercises, or orthotic support when clinically indicated. In contrast, rigid flatfoot should always prompt further evaluation because it may indicate underlying pathology, including tarsal coalition, congenital vertical talus, inflammatory conditions, or other structural abnormalities. Painful flatfoot associated with a prominent accessory navicular should also be considered in older children and adolescents. These patients should be referred for orthopaedic assessment and appropriate imaging.

Clinical examination should include assessment of hindfoot flexibility, restoration of the medial longitudinal arch during tiptoe standing or the single heel-rise test, and subtalar joint motion. The presence of a “too many toes” sign, medial prominence over the navicular bone suggestive of an accessory navicular, localized tenderness, or subtalar stiffness may indicate underlying pathology and warrants further evaluation or referral to a paediatric orthopaedic specialist [49,50].

### 5.6. Management of Rotational Deformities

Conservative management consists primarily of monitoring growth patterns, as intervention is rarely required in physiologically developing children. Treatment is rarely indicated, except in severe, symptomatic cases persisting beyond the age of 8 years, or when associated with underlying conditions such as cerebral palsy or SCFE [9,11].

### 5.7. Summary of Rotational Deformities

Rotational alignment variations are very common in childhood and usually resolve with growth. Persistent, unilateral, or symptomatic cases, particularly those associated with neuromuscular disorders or slipped capital femoral epiphysis (SCFE), should prompt further evaluation and referral. In clinical practice, careful history, physical examination, and regular follow-up are usually sufficient to distinguish physiological from pathological findings.

## 6. Follow-Up

In the absence of red flags, structured clinical follow-up is sufficient, as intervention is rarely indicated in physiologically developing children. In children whose clinical findings are consistent with physiological development, structured clinical follow-up at 6–12-month intervals is sufficient to monitor growth and alignment. In cases with atypical or progressive features, shorter follow-up intervals (every 3–6 months) are appropriate.

Conservative interventions such as orthotics, shoe inserts, or exercises have no proven effectiveness in physiological cases and are therefore not routinely recommended [2,24,47,48,51,52].

**Figure 8:**
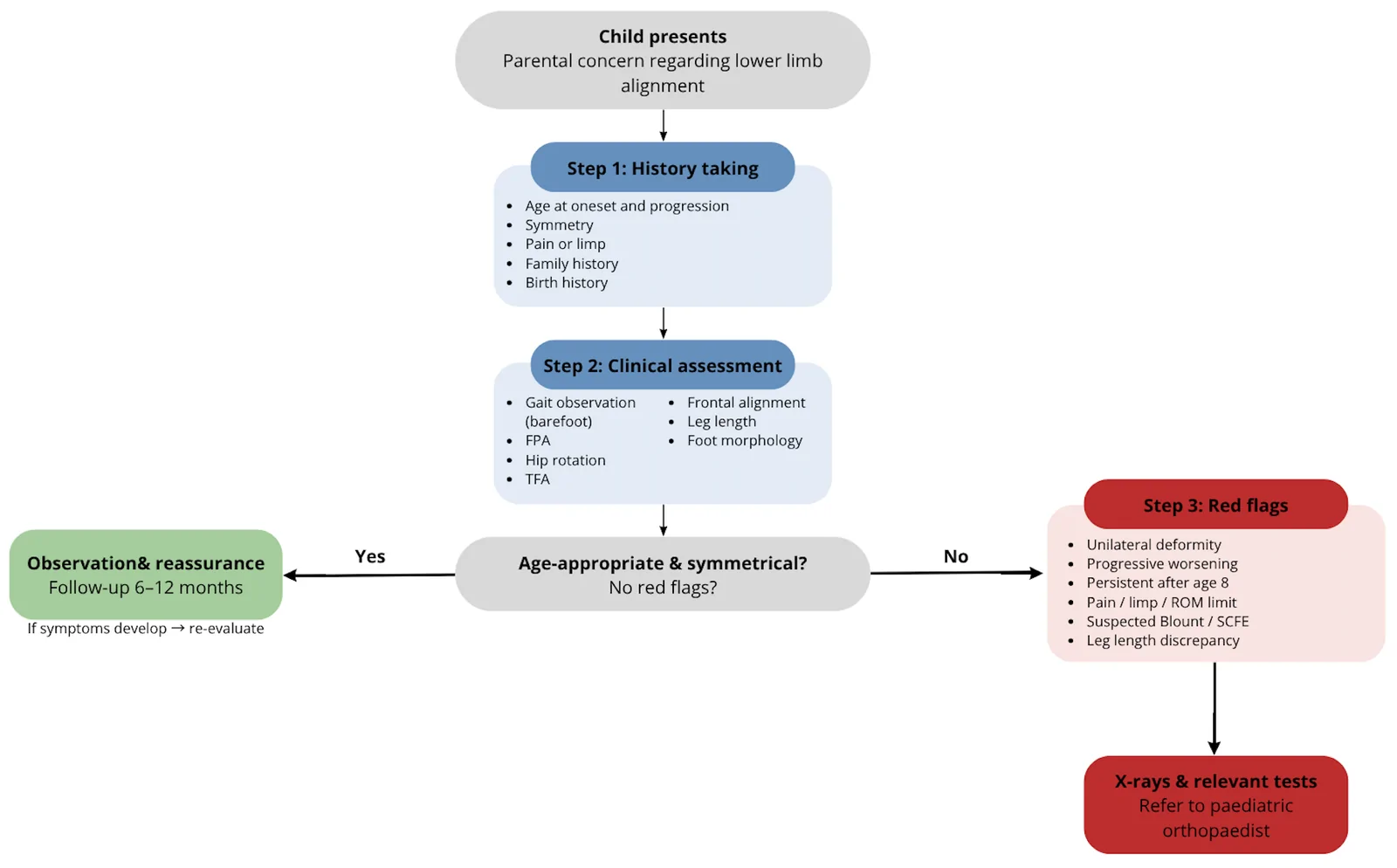
Clinical algorithm for evaluation of lower limb alignment in children.

Thus, while most cases require only reassurance and observation, careful monitoring ensures timely recognition of the minority of children who need specialist intervention. A structured clinical algorithm for the evaluation of lower limb alignment variations in children is presented in Figure 8.

**Indications for Referral to a Paediatric Orthopaedist**

While the majority of cases involving lower limb alignment in children require only reassurance and counselling of parents, certain situations necessitate specialist evaluation. Paediatricians should recognize when the clinical course deviates from normal developmental patterns and refer appropriately. Referral to a paediatric orthopaedic surgeon is warranted in the following situations:

• Unilateral or asymmetrical deformities,
• Lack of improvement or progression after the age of 8 years,
• Presence of pain, limping, or restricted range of motion,
• Positive family history of skeletal disorders,
• Leg length discrepancy,
• Neurological signs or symptoms,
• Suspected pathological conditions such as Blount’s disease, rickets, SCFE, or congenital limb abnormalities.

Timely referral in these situations ensures early diagnosis and appropriate management, reducing the risk of long-term complications.

A comprehensive overview of lower extremity conditions in children, including typical presentation, diagnostic criteria, red flags, and management, is provided in Table 5.

## 7. Key Clinical Take-Home Points

1. Most lower limb alignment concerns in children represent physiological developmental variations rather than true deformities.
2. Coronal alignment typically progresses from infant genu varum to transient genu valgum (3–6 years), stabilizing by 7–8 years.
3. In-toeing is most commonly caused by femoral anteversion, internal tibial torsion, or metatarsus adductus and usually resolves spontaneously.
4. Out-toeing is less frequently physiological and warrants careful evaluation, particularly in adolescents.
5. Red flags include asymmetry, progression, pain, limping, restricted range of motion, leg length discrepancy, or neurological findings.
6. Suspected SCFE requires urgent radiographic assessment and specialist referral, especially in adolescents presenting with:
  • new-onset out-toeing,
  • limp,
  • hip or referred knee pain,
  • limited internal rotation of the hip,
  • obligate external rotation during hip flexion.
7. In the absence of red flags, structured clinical follow-up is sufficient, orthotics are not beneficial, and intervention is rarely indicated.

## 8. Conclusions

Parental concerns regarding lower limb alignment and rotation are among the most common reasons for paediatric consultations; however, in the vast majority of children, these findings represent physiological developmental variations rather than true deformities. A thorough understanding of age-appropriate developmental norms, combined with systematic clinical assessment—including gait observation, foot progression angle, hip rotation, and thigh–foot angle—enables paediatricians to accurately differentiate benign variants from conditions requiring intervention.

Conservative management consists primarily of monitoring growth patterns and parental reassurance. Orthotics, shoe inserts, and other conservative interventions have no proven efficacy in physiological cases and are therefore not routinely recommended. Intervention is rarely indicated in physiologically developing children.

Recognition of red flags—including asymmetry, progression beyond expected age ranges, pain, limping, restricted range of motion, leg length discrepancy, or neurological signs—is essential for timely specialist referral. Particular vigilance is warranted in adolescents presenting with new-onset out-toeing, hip pain, or restricted internal rotation, given the risk of slipped capital femoral epiphysis.

Future advances in digital gait analysis and automated video-based assessment tools may further support early differentiation between physiological variants and pathological conditions in primary care settings.

**Figure 9:**
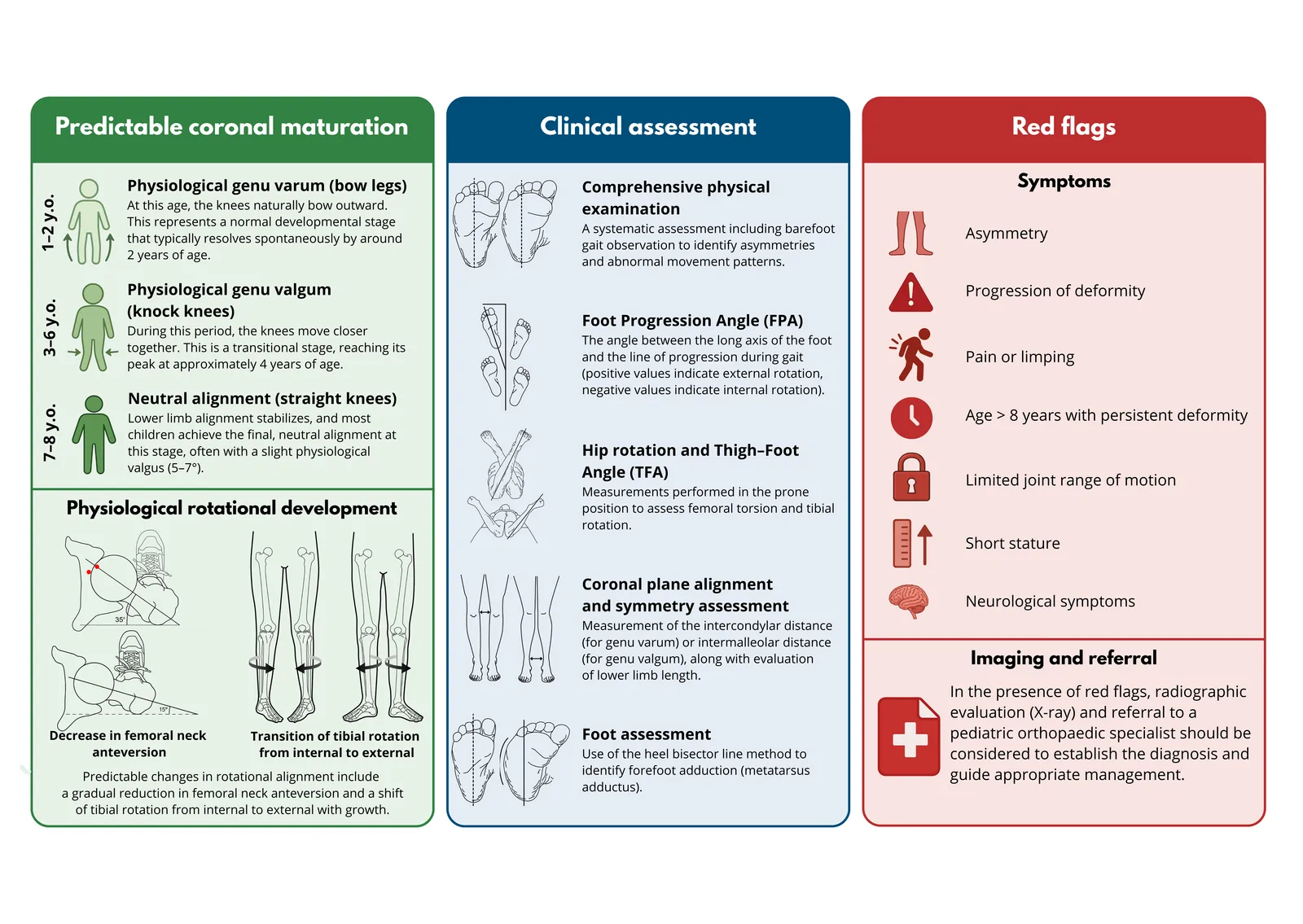
Assessment of lower limb alignment in children: physiological development versus pathological indicators.

A comprehensive graphical summary of physiological development, clinical assessment, and red flags is presented in Figure 9.

## Figures and Tables

**Table 3:** Physical examination in children with lower extremity alignment or rotational abnormalities.

Examination Component	How to Perform	Key Findings	Possible Differential Diagnoses
Gait observation	Observe the child walking and running in a straight line. Evaluate foot progression angle and overall limb alignment.	In-toeing, out-toeing, limp, asymmetric stride	Femoral anteversion, tibial torsion, metatarsus adductus, SCFE, neuromuscular disorders
Foot progression angle (FPA)	Angle between the long axis of the foot and the direction of walking.	Negative values: in-toeing; Positive values: out-toeing	Femoral anteversion, tibial torsion, femoral retroversion
Thigh–foot angle (TFA)	Child prone with knees flexed to 90°. Compare the axis of the thigh with the axis of the foot.	Negative angle: internal tibial torsion; Positive angle: external tibial torsion	Internal tibial torsion, external tibial torsion
Hip rotation	Child prone, knees flexed to 90°. Measure internal and external rotation of the hips.	Increased internal rotation (>70°) suggests femoral anteversion; limited internal rotation may indicate pathology	Femoral anteversion, SCFE, developmental dysplasia of the hip
Heel bisector line	Draw an imaginary line from the heel through the forefoot while the child is standing or supine.	Line passing lateral to second toe indicates metatarsus adductus	Metatarsus adductus
Intercondylar distance	With ankles together, measure distance between medial femoral condyles.	Increased distance suggests genu varum	Physiologic bowlegs, Blount disease, rickets
Intermalleolar distance	With knees together, measure distance between medial malleoli.	Increased distance suggests genu valgum	Physiologic genu valgum, skeletal dysplasia
Limb length comparison	Compare limb lengths clinically and assess pelvic tilt.	Limb length discrepancy	Growth plate injury, congenital limb abnormalities
Foot flexibility	Attempt passive correction of forefoot alignment.	Flexible deformity vs rigid deformity	Flexible metatarsus adductus vs rigid deformity
Neurological examination	Assess muscle tone, reflexes, and coordination.	Hypertonia, hypotonia, abnormal reflexes	Cerebral palsy, neuromuscular disorders

**Table 4:** Age-related normative values for rotational alignment of the lower limb. Values represent approximate clinical reference ranges derived from observational and cross-sectional studies and should be interpreted in conjunction with symmetry and overall development.

Parameter	Newborn	Age 2–3 Years	Age 5–6 Years	Adolescence
**Femoral anteversion**	Up to 40°	~30–35°	~25°	~15°
**Tibial torsion**	−5° (internal)	0°	+10° (external)	Up to +25°
**Thigh–foot angle (TFA)**	−5°	0°	+10°	+10–15°
**Foot progression angle (FPA)**	Variable	Slight internal	Neutral to +5°	+5° to +10°
**Metatarsus adductus angle**	0–15°	0–15°	0–15°	0–15°

**Table 5:** Summary of lower extremity conditions in children.

Condition	Epidemiology/Typical Age	Presentation	Diagnostic Criteria/Key Examination Findings	Red Flags Suggesting Pathology	Management
Physiological genu varum	Birth–2 years	Symmetrical bowlegs	Bilateral age-appropriate varus alignment	Persistence after 2–3 years, asymmetry, progression, pain	Observation, reassurance
Pathological genu varum	>2 years or progressive	Unilateral/progressive bowing	Increased intercondylar distance	Rapid progression, unilateral deformity, pain	Orthopaedic referral
Physiological genu valgum	3–6 years	Symmetrical knock knees	Age-appropriate intermalleolar distance	Persistence after ~8 years, asymmetry	Observation
Pathological genu valgum	Beyond expected age	Knock knees with symptoms	Excessive intermalleolar distance	Progressive deformity, gait disturbance	Orthopaedic referral
Metatarsus adductus	Infancy	Curved lateral foot border	Heel bisector deviation	Rigid deformity, persistence >1 year	Observation or casting
Internal tibial torsion	1–3 years	Intoeing gait	Negative thigh–foot angle	Persistence after ~8 years, asymmetry	Observation
Femoral anteversion	3–6 years	Intoeing, W-sitting	Increased internal hip rotation	Persistence after 8–10 years, functional limitation	Observation or surgery
Out-toeing	Childhood/adolescence	Feet point outward	Positive foot progression angle	New onset with pain or limp	Evaluate rotational profile
External tibial torsion	Later childhood	Out-toeing gait	Positive thigh–foot angle	Severe deformity, patellofemoral symptoms	Referral if symptomatic
Femoral retroversion	Infancy	Out-toeing	Increased external hip rotation	Persistence beyond early childhood	Referral
Pes planus (flexible)	Early childhood	Flattened arch	Arch reappears on tiptoe	Pain, stiffness, rigid flatfoot	Reassurance

## Data Availability

No new data were created or analyzed in this study. Data sharing is not applicable to this article.
